# Phytochemical Profile of *Convolvulus cantabrica* Extracts and Their Biological Activity

**DOI:** 10.3390/molecules31010058

**Published:** 2025-12-23

**Authors:** Khaled Ben Elwalid Mahdadi, Zina Allaoua, Mohamed Sabri Bensaad, Fatima Belahssini, Chawki Bensouici, Diana C. G. A. Pinto, Yavuz Selim Cakmak, Hamada Haba, Dimitris Mossialos, Dimitrios Stagos, Salah Akkal

**Affiliations:** 1 Laboratory of Chemistry and Environmental Chemistry (L.C.C.E), Department of Chemistry, Faculty of Science, University of Batna 1, Batna 05000, Algeria; khaledbenelwalid.mahdadi@univ-batna.dz (K.B.E.M.); zina.allaoua@univ-batna.dz (Z.A.); fatima.belahssini@univ-batna.dz (F.B.);; 2 Department of Biology of Organisms, Faculty of Natural and Life Sciences, University Batna 2, Fesdis, Batna 05078, Algeria; 3 Biotechnology Research Center, Constantine 25000, Algeria; 4 LAQV-REQUIMTE and Department of Chemistry, University of Aveiro, 3810-193 Aveiro, Portugal; diana@ua.pt; 5 Department of Biotechnology and Molecular Biology, Faculty of Science and Arts, Aksaray University, Aksaray 68100, Turkey; yavuzselimcakmak@gmail.com; 6 Department of Biochemistry and Biotechnology, School of Health Sciences, University of Thessaly, Biopolis, 41500 Larissa, Greece; mosial@bio.uth.gr; 7 Valorization of Natural Resources, Bioactive Molecules and Biological Analysis Unit, Department of Chemistry, Frères Mentouri Constantine 1 University, Constantine 25000, Algeria

**Keywords:** *Convolvulus cantabrica*, polyphenols, antioxidant, antibacterial, anti-inflammatory

## Abstract

The present work provides a detailed study of *Convolvulus cantabrica* L., a plant belonging to the family Convolvulaceae and the genus *Convolvulus*. The selection of this plant was based on the long-standing ethnobotanical relevance of its genus, which was attributed to the richness of its species in phenolic and flavonoids compounds. Moreover, this species as remained unexplored to date. Our investigation includes both chemical and biological aspects. To assess the chemical composition of the hydroalcoholic extract of the plant, High-Performance Liquid Chromatography (HPLC) analysis was performed. Rosmarinic Acid (161.9 ppm) and Chlorogenic Acid (153.8 ppm) had the highest concentrations. Gas Chromatography–Mass Spectrometry (GC-MS) analysis demonstrated the presence of Fatty Acids and Esters (70.81%), sesquiterpene and diterpenes (19.51%) and fatty alcohols (6.02%). In addition, the ethyl acetate extract exhibited the highest phenolic contents (606.42 µg/mL) and flavonoid contents (363.75 µg/mL). The tested extracts, especially the ethyl acetate and butanol extracts, exhibited strong antioxidant capacity in DPPH (IC_50_: 13.60 ± 1.30 µg/mL for ethyl acetate extract and 17.69 ± 1.17 µg/mL for butanol extract), ABTS (IC_50_: 7.26 ± 0.01 µg/mL for ethyl acetate extract and 6.90 ± 0.18 µg/mL for butanol extract) and FRP (IC_50_: 14.89 ± 0.90 µg/mL for ethyl acetate extract and 23.14 ± 0.60 µg/mL for butanol extract) assays compared with extracts from other species of this genus. Moreover, the petroleum ether extract demonstrated anti-inflammatory activity (IC_50_: 419.30 ± 4.48 µg/mL). Regarding antibacterial activity, the plant extracts, especially the ethyl acetate, hydroalcoholic and petroleum ether extracts, inhibited the growth of *Bacillus cereus*. Overall, our data indicate that *Convolvulus cantabrica* L., is rich in secondary metabolites, particularly polyphenols, and exhibits significant biological activities, especially antioxidant properties. These results validate the traditional use of *C*. *cantabrica* and position it as a promising source of natural antioxidants with potential pharmaceutical and nutraceutical applications.

## 1. Introduction

Given the global rise in antimicrobial resistance, chronic inflammation, and oxidative stress-related diseases, there is a growing and urgent need to discover new and effective bioactive molecules. Therefore, scientists are increasingly focusing on the study of medicinal plants, which represent a valuable reservoir of such compounds. From ancient times to the present day, herbs have been officially considered to be an effective treatment for many diseases [1]. The diversity of medicinal plants used reflects the ongoing search for effective and less toxic therapeutic alternatives. Chemists are striving to isolate and purify various secondary metabolites and then to evaluate their biological activities. Plant-derived natural products have the potential to reduce oxidative stress [2] and to support and improve traditional medicines. Such compounds have made a significant impact on the treatment of various human diseases, emphasizing the importance of this field in advancing humanity’s efforts in medicine [3].

Besides its various traditional uses, the Convolvulaceae family has been attracted pharmacological interest due to its secondary metabolites and bioactive compounds. Several genera within this family have shown antioxidant, anti-inflammatory, antitumor, and antispasmodic activities [4,5]. The major classes of secondary metabolites include alkaloids, flavonoids, coumarins, sterols, saponins, tannins, and various other isolated compounds [4,6,7].

Given its extensive area and diverse climate, Algeria possesses a wide variety of plant life. The flora of Algeria is characterized by the prevalence of flowering plants within the desert ecosystem, comprising some 2500–3000 species and infraspecific taxa [8], including 653 endemics species. However, despite this remarkable plant biodiversity, it has been explored from chemical and pharmacological perspectives only to a limited extent. Over the years, advances in chemical investigation methods—and particularly in the isolation of biologically active compounds—have enabled scientists to identify an increasing number of secondary metabolites and bioactive compounds with potential therapeutic value.

Within Algeria’s rich botanical heritage, the Convolvulaceae family occupies an important place due to its wide distribution across various ecosystems and its traditional medicinal value. This context highlights the scientific interest in exploring this family [9]. Specifically, the Convolvulaceae family consists of 60 genera and approximately 2000 species [10]. Its distribution is cosmopolitan, with a particular prevalence in warm and temperate regions. Species within this family are predominantly herbaceous—often climbing—along with some shrubs and subshrubs [10,11]. Among the morphological characteristics of this family are the presence of laticiferous cells, bicollateral vascular bundles, and often abnormal vascular patterning. These characteristics are particularly prominent in medicinal plants, including species of *Ipomoea*. For instance, jalap refers to the starch extracted from the tuberous roots of sweet potato (*Ipomoea batatas*) [4,12]. Several chemical studies have investigated the bioactive constituents of this plant family, identifying compounds such as indole, isoquinoline, pyrrolidine, tropane alkaloids, purgative resins, phenolic acids, and triterpenoid saponins [13].

The genus *Convolvulus* (commonly known as bindweeds), whose name is derived from the Latin verb *convolvere*, meaning “to entwine” or “to coil”, refers to the characteristic twining habit of its stems around supporting structures. It is one of the principal genera within the Convolvulaceae family, comprising approximately 203 species. This places it as the third most diverse genus in the family, following *Ipomoea* (615 species) and *Cuscuta* (220 species) [14]. The *Convolvulus* genus has a cosmopolitan distribution, with most of its species occurring in the Mediterranean region and Western Asia [15]. In Algeria, this genus is represented by many species. Many species of the *Convolvulus* genus are known for their medicinal value and diverse biological activities, including purgative, central nervous system-disrupting and antidepressant effects, as well as antioxidant, hypoglycemic, antinociceptive, anticancer, antiulcer, and antidiarrheal properties [16,17,18,19,20]. In this context, a specific species, *Convolvulus cantabrica*, which is primarily found in alkaline soils and sunny environments [21], has been traditionally used to treat coughs, skin conditions, and various inflammatory disorders related to fever and joint pain [19]. However, to date, no chemical or biological studies have been conducted. For these reasons, the present study aimed, for the first time, to evaluate both the phytochemical composition and the pharmacological properties of *Convolvulus cantabrica* using various analytical and biological methods.

## 2. Results and Discussion

### 2.1. Liquid-Liquid Extraction

The weight and the percentage yield of the three extracts obtained from *Convolvulus cantabrica* are presented in Table 1.

### 2.2. Total Phenolic, Flavonoid and Flavonol Contents

The results of total phenolic and flavonoid contents are presented in Table 2. The ethyl acetate extract exhibited the highest flavonoid content (363.75 µg/mg) and phenolic content (606.421 µg/mg), followed by the n-butanol extract (221.527 and 414.460 μg/mg, respectively). In contrast, the hydro-alcoholic extract showed the lowest flavonoid content (65.277 µg/mL), while the petroleum ether extract had the lowest phenolic content (67.009 µg/mL). Flavonol content analysis revealed that the ethyl acetate extract also had the highest flavonol content (127.441 µg/mg), followed by the petroleum ether extract (95.328 µg/mg) and the n-butanol extract (93.075 µg/mg). Notably, the hydro-alcoholic extract again exhibited the lowest flavonol content (32.253 µg/mg).

Phenolic compounds are among the most abundant secondary metabolites in plants and are known for their significant pharmacological effects on the human body. These include protective mechanisms against various stress factors [22]. The total phenolic content in *Convolvulus cantabrica* L. extracts was determined using solvents of increasing polarity, from non-polar to highly polar. The quantitative analysis revealed variation in polyphenol and flavonoid levels based on the polarity of the extracting solvent. Among the tested extracts, ethyl acetate and n-butanol extracts exhibited the highest concentrations of polyphenols and flavonoids. Remarkably, the levels observed in these extracts exceeded those reported by Al-Rifai et al. [23] for *Convolvulus pilosellifolius* and by Osman et al. [16] for *Convolvulus arvensis*. These findings highlight the high efficiency of solvent polarity in the extraction process [24] as well as the influence of other factors such as extraction methods, solvent-plant interactions, and the precision of extraction techniques.

### 2.3. Identification of Polyphenolic Composition of Hydroalcoholic Extract by HPLC

HPLC analysis of hydroalcoholic extract revealed the presence of 7 phenolic compounds: Chlorogenic Acid, Caffeic Acid, Gallic Acid, Hesperidin, Quercetin, Rosmarinic Acid, trans-Cinnamic Acid (Table 3). Notably, the most abundant phenolic acids identified were rosmarinic acid (161.897 ppm). Rosmarinic acid (161.897 ppm) is known for its broad range of biological activities, including anti-diabetic, anticancer, anti-inflammatory, and antioxidant effects [25]. Chlorogenic acid (153.765 ppm) has been implicated in multiple chronic metabolic and age-related disorders. Current evidence demonstrates its wide biological activities, including neuroprotection relevant to neurodegenerative diseases and diabetic peripheral neuropathy, as well as anti-inflammatory, antioxidant, antimicrobial, cardioprotective, dermatoprotective, antidiabetic, hepatoprotective, nephroprotective, and antitumor properties [26]. Caffeic acid (33.047 ppm) also exhibits multiple biological activities, such as potent antioxidant action, anticancer effects through the inhibition of cell proliferation and induction of apoptosis, anti-diabetic activity via improved insulin sensitivity and metabolic regulation, anti-obesity effects through reduced lipid accumulation, and neuroprotective activity that mitigates oxidative stress and neuroinflammation in disorders such as Alzheimer’s and Parkinson’s disease [27]. Among the flavonoids, hesperidin was the most prevalent (7.459 ppm), followed by quercetin (3.047 ppm) (Table 3). Other compounds, such as trans-Cinnamic Acid and Gallic acid, were present in smaller amounts. Several other compounds were not detected (N.D.), likely due to their concentrations being below the detection limit or their absence in the sample.

Previous HPLC analyses of various *Convolvulus* species have revealed the presence of a wide range of phenolic acids, flavonoids, terpenoids, and polysaccharide-type compounds [19,28,29]. For instance, phytochemical screening of *Convolvulus betonicifolia* identified similar phenolic acids, including Rosmarinic acid, Caffeic acid, Chlorogenic acid, and syringic acid; however, the concentrations observed in our study were significantly higher. Similarly, comparable flavonoids such as quercetin, hesperidin, and epicatechin were reported in *C. betonicifolia* [28]. Moreover, HPLC analysis of the crude extract of *Convolvulus lineatus* confirmed the presence of Catechin, hesperidin, and other flavonoid sulfates and flavone-based compounds [30], which aligns with the findings of the current study. It is important to note that a high concentration of these phytochemicals has been previously associated with potent antioxidant and anti-inflammatory activities. The results of our in vitro biological capacity assays (DPPH, ABTS, FRP, etc.) support the presence of significant bioactive compounds, correlating well with the phytochemical content identified [31]. The corresponding HPLC chromatogram is provided in the Supplementary Materials Figure S1.

### 2.4. Compounds Identified in Petroleum Ether Extract by GC-MS Analysis

The petroleum ether extract was dominated by volatile and semi-volatile compounds, which are suitable for analysis under GC-MS conditions, whereas the three other extracts contain non-volatile or thermolabile metabolites unsuitable for direct GC-MS characterization.

**Figure 1:**
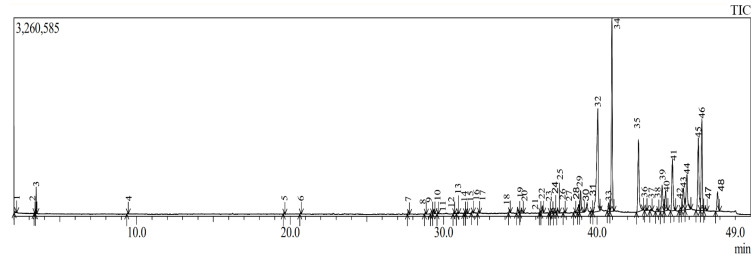
Chromatogram of GC-MS analysis of petroleum ether extract.

The petroleum ether extract was obtained through maceration in an alcoholic medium followed by liquid–liquid extraction based on polarity. GC–MS analysis of this extract identified 48 chemical compounds belonging to various chemical families (Table 4; Figure 1). Among the identified compounds, fatty acids and esters were the most predominant, representing 70.81% of the total composition (Table 4; Figure 1). Additionally, compounds of sesquiterpene and diterpene nature accounted for 19.51% (Table 4; Figure 1). The remaining chemical families were present in smaller proportions. The extract was found to be particularly rich in fatty acid esters, fatty acids, sesquiterpene alcohols, alkenes, fatty alcohols, ketones, and aromatic hydrocarbons. The richness in these classes of secondary metabolites is noteworthy, as such compounds are often associated with various biological activities, including antioxidant, anti-inflammatory, and antimicrobial effects [32,33]. A similar GC–MS analysis conducted by Hrichi et al. [34] on *Convolvulus althaeoides* identified 86 chemical compounds, with n-hexadecanoic acid and linoleic acid found in the highest proportions (29.77% and 7.30%, respectively), which is in agreement with our findings Furthermore, another study on *C. althaeoides* reported the presence of sesquiterpene hydrocarbons, oxygenated sesquiterpenes, and oxygenated monoterpenes, including germacrene D, τ-cadinol, and verbenone. This partially aligns with our data, as a non-negligible proportion of sesquiterpene and diterpene compounds were also identified in our extract [35].

### 2.5. Antioxidant Capacity

The results of the 2,2-diphenyl-1-picrylhydrazyl (DPPH) assay, presented in Table 5, revealed that among the four tested extracts, the ethyl acetate extract exhibited the strongest DPPH radical scavenging capacity, with an IC_50_ value of 13.60 ± 1.30 µg/mL, which was significantly better (*p* < 0.001) than the tested standard BHT (22.32 ± 1.19 µg/mL) (Table 5). Notably, this extract also showed remarkable reducing power capacity, with an A_0.5_ value of 14.89 ± 0.90 µg/mL, which was significantly greater than that of the standard α-tocopherol (Table 5).

In the 2,2′-azino-bis(3-ethyl-benzothiazoline-6-sulfonic acid (ABTS^•+^) assay, the n-butanol extract exhibited the strongest capacity (6.90 ± 0.18 µg/mL), followed closely by the ethyl acetate extract (7.26 ± 0.01 µg/mL) (Table 5). The differences between these and the reference antioxidants butylated hydroxyanisole (BHA) and butylated hydroxytoluene (BHT) were highly significant (Table 5).

Antioxidants play a critical role in protecting biological tissues from oxidative damage caused by free radicals through various mechanisms [36]. As antioxidant responses can vary depending on the assay system used, numerous analytical methods have been developed to evaluate the antioxidant capacity of plant extracts based on their polarity. These include direct and indirect assays, competitive procedures, and methods based on reduction mechanisms, mineral chelation, or inhibition processes [16]. It is well established that polyphenols, flavonoids, and flavonols possess antioxidant capacity, and their effectiveness correlates with their concentration; that is, higher levels result in stronger antioxidant effects. In this study, the ethyl acetate and n-butanol extracts demonstrated notably high antioxidant capacity. This is likely due to their high content of polyphenols and flavonoids [37], which contain multiple hydroxyl groups, double bonds, and free electrons that facilitate hydrogen or electron donation to neutralize reactive species [38].

The genus *Convolvulus* has been previously shown to exhibit strong antioxidant capacity, particularly against synthetic free radicals such as DPPH and ABTS^•+^. Our findings further confirm this, as both ethyl acetate and n-butanol extracts demonstrated significant antioxidant capacity that was greater than those reported for other *Convolvulus* species. For example, Thakral et al. [39] reported that methanolic and ethyl acetate fractions of Convolvulus arvensis exhibited dose-dependent radical scavenging capacity in both DPPH and ferric reducing antioxidant power assay (FRP) assays, attributed to their phenolic acid and flavonoid content. Similarly, phytochemical screening of the aerial parts of *Convolvulus prostratus* identified gallic acid, quercetin, rutin, rosmarinic acid, trans-cinnamic acid, and chlorogenic acid, which are known for their antioxidant effects. These bioactive compounds are believed to be effective scavengers of superoxide anions and hydrogen peroxide, by enhancing intracellular glutathione peroxidase, inhibiting NADPH oxidase, and reducing lipid peroxidation [20,23,39,40]. Importantly, many of these same compounds were also detected in our study.

Moreover, the ethyl acetate and n-butanol extracts from *C. cantabrica* demonstrated a strong ability to reduce ferric ions, which is indicative of their capacity to regenerate oxidized molecules via electron donation. The reducing power assay, used in this study, measures the transformation of Fe^3+^(CN)_6_ to Fe^2+^(CN)_6_, reflecting the direct electron-donating ability of the extract [20]. In support of our results, *Convolvulus phrygius* also exhibited significant antioxidant capacity in its ethyl acetate extract and at similar extract concentrations to our study, as shown using ABTS^•+^, NO, FRP, phosphomolybdenum, and metal-chelating assays [24]. Thus, these findings are in strong agreement with our study.

### 2.6. Anti-Inflammatory Activity

Only the petroleum ether extract exhibited notable anti-inflammatory activity (IC_50_ 419.30 ± 4.48 µg/mL). The IC_50_ value of the ethyl acetate extract in the anti-inflammatory assay was 3172.65 ± 35.05 µg/mL. The other extracts did not show any activity (Table 6). However, this anti-inflammatory effect was significantly (*p* < 0.001) lower than that of the standard diclofenac sodium (Table 6). The search for safe, plant-based sources to treat inflammatory disorders has intensified due to the adverse effects associated with synthetic drugs. Previous studies have shown that several plant species belonging to the Convolvulaceae family possess notable anti-inflammatory properties. Among them, *Convolvulus cantabrica* demonstrated promising results, particularly with the petroleum ether extract, likely due to its content of terpenes and other bioactive chemical families known for their positive biological effects [41]. In addition, similar phenolic acids and flavonoids have been identified in *Convolvulus arvensis*, where studies revealed its ability to suppress pro-inflammatory cytokine production, such as IL-6, TNF-α, and MCP-1, along with inflammatory enzymes like COX-2 and iNOS [20]. Furthermore, the presence of hydroxycinnamic acid and trans-cinnamic acid in *Convolvulus pluricaulis* may explain its ability to attenuate neuroinflammation, a finding supported by studies using a chronic rat model of depression [42]. In our study, rosmarinic acid was identified in high concentrations, and this phenolic acid has been reported to significantly reduce abnormal eosinophil and neutrophil levels during immune responses. It also lowers the expression of inflammatory cytokines such as IL-4, IL-5, and eotaxin through the activation of the Nrf2 signaling pathway [43].

### 2.7. Antibacterial Activity

The antibacterial activity of four plant extracts implementing solvents of different polarities against four bacterial species was assessed by measuring the diameter of the inhibition zones compared to positive control (Imipenem) (Table 7). A significant antibacterial activity has been exerted by the three *C. cantabrica* extracts against the Gram-positive *Bacillus cereus*, a foodborne pathogen implicated in food intoxications. Regarding the petroleum ether extract, inhibition zones ranged from 6.5 to 9 mm, depending on the tested concentration (Table 7). These inhibition zones were significantly lower than those of the positive control Imipenem (40.1 mm), indicating that the extract was less effective (Table 7). The ethyl acetate extract demonstrated moderate antibacterial activity, with the highest inhibition zone measuring 10.5 mm at a concentration of 100 mg/mL, while lower inhibition values were observed at lower concentrations (Table 7). Similarly, the hydroalcoholic extract showed a comparable highest inhibition zone (10.5 mm) at the same concentration, followed by declined inhibition zones at lower doses (Table 7). However, *C. cantabrica* extracts did not exhibit antibacterial activity against the other three tested bacterial species, that is, *E. coli*, *S. aureus* and *S. epidermidis* (Table 7).

Although the extracts were tested at a relatively high concentration (100 mg/mL), they exhibited a limited antibacterial effect, particularly against Gram-positive strains. This narrow spectrum could be explained by the chemical composition of the extracts, in which bioactive compounds are present in insufficient quantities or are less effective against certain bacteria. Additionally, the diffusion of lipophilic constituents in agar may be limited, reducing the efficacy of the petroleum ether extract. These results suggest that while *C. cantabrica* extracts possess antibacterial potential, their efficacy is strain-dependent and may require further fractionation or the use of alternative extraction solvents to achieve broader activity. This could explain why activity is weaker or absent in certain strains. Interestingly, studies on *Convolvulus arvensis* reported similar antibacterial activity against *Bacillus cereus*, attributed to the presence of terpenes, terpenoids, and phenolic compounds [20]. Additionally, a study by Al-Rifai et al. [23] demonstrated the antimicrobial activity of *Convolvulus pilosellifolius* and *Convolvulus austroaegyptiacus*, which was attributed to fatty acids, alkaloids, flavonoids, and phenolic compounds. HPLC analysis revealed the presence of phenolic compounds, and the total phenolic, flavonoid, and flavonol contents were high, which may explain the observed antibacterial effects.

## 3. Materials and Methods

### 3.1. Chemicals and Standards

All solvents, standards and culture media used in this study were purchased from Sigma Aldrich (St. Louis, MO, USA): 1,4-bis(dimethylsiloxy)-phenylenedimethylpolysiloxane, 2,2′-azinobis-(3-ethylbenzothiazoline)-6-sulfonic acid, 2,2-diphenyl-1-picrylhydrazyl, 3-hydroxy benzoic acid, 4-hydroxy benzoic acid, acetonitrile, aluminum chloride, benzoic acid, butylated hydroxyanisole, butylated hydroxytoluene, caffeic acid, catechin hydrate, chlorogenic acid, cinnamic acid, dimethyl sulfoxide, epicatechin, ethyl acetate, folin-ciocalteu, gallic acid, hesperidin, methanol, Mueller-Hinton agar, n-butanol, p-coumaric acid, petroleum ether, phosphoric acid, potassium ferricyanide, rosmarinic acid, quercetin, sinnapic acid, sodium acetate, sodium carbonate, syringic acid, t-cinnamic acid, t-ferulic acid, trichloroacetic acid and tris hydrochloride.

### 3.2. Plant Material

The plant was collected in June 2023 from the mountains of Merouana (Batna, Algeria; 35°35041.5200 N, 5°56013.7500 E). Its authenticity was confirmed by Professor Bachir Oudjihih, Agronomic Institute of the University of Batna-1 (Algeria), according to the description provided under reference number LCCE/1120. The specimen has been preserved at the herbarium of the Chemistry and Environmental Chemistry Laboratory.

### 3.3. Maceration and Extraction of Secondary Metabolites

A total of 700 g of dried plant material was finely ground and subjected to maceration in a 70% ethanol-water solution repeated three times for 72 h each on the same plant material (72 × 3) under continuous agitation. The resulting mixture was filtered and evaporated at 35 °C to obtain the hydroalcoholic extract. From the final extract, 5 g were stored for further use in the biological activity assays, while the remaining portion was used in the liquid–liquid extraction.

To separate secondary metabolites based on their polarity, a liquid–liquid extraction was performed using three solvents of increasing polarity: petroleum ether (for non-polar compounds), ethyl acetate (for medium-polar compounds), and n-butanol (for polar compounds).

The hydroalcoholic extract was first suspended in 100 mL of distilled water and then extracted three times with petroleum ether (300 mL each) using a separating funnel. The aqueous phase was subsequently partitioned with ethyl acetate (300 mL each) and then extracted with n-butanol (300 mL each). Each organic phase was collected, dried over anhydrous sodium sulfate, filtered, and evaporated to obtain the respective fraction.

### 3.4. HPLC of the Hydroalcoholic Extract

Sample preparation was performed by dissolving 10 mg of the extract in 1 mL of HPLC-grade methanol. The mobile phase consisting of water containing 0.1% phosphoric acid (A) and 100% acetonitrile (B). The elution program was carried out using the following gradient: 0–6 min: 83% A/17% B; 7–19 min: 85% A/15% B; 20–23 min: 80% A/20% B; 24–27 min: 75% A/25% B; 28–29 min: 70% A/30% B; 30–31 min: 60% A/40% B; 32–35 min: 50% A/50% B; 36–39 min: 30% A/70% B. The flow rate was set at 0.80 mL/min, with an injection volume of 10 μL. The column temperature was maintained at 20 °C. Detection wavelengths were set at 200 nm and 300 nm.

Identification of compounds was accomplished by comparing the retention times and UV spectra of sample peaks to those of authentic standards (16 compounds). The mixed standard solution was prepared by diluting the stock solutions of each polyphenol in methanol to a final concentration of 16 mg/L (16 ppm) for each compound. This assay method was adapted from Ayad et al. [44].

### 3.5. Gas Chromatography–Mass Spectrometry (GC–MS) Analysis of the Petroleum Ether Extract

Gas chromatography-mass spectrometry (GC-MS) was used to separate and identify the chemical constituents of the petroleum ether extract. The analysis was performed on a Shimadzu GC-MS-QP2020 system equipped with an automatic injector (AOC-20i) (Shimadzu Corporation, Kyoto, Japan) [28]. This separation was carried out using a fused silica capillary column (Rxi-5Sil MS, Restek Corporation, Bellefonte, PA, USA), 30 m in length, with an internal diameter of 0.25 mm and a film thickness of 0.25 μm. The stationary phase consisted of cross-bonded 1,4-bis(dimethylsiloxy)-phenylenedimethylpolysiloxane, a low-polarity, chemically inert, and thermally stable material suitable for operation over a wide temperature range (−60 °C to 350 °C). This phase is chemically similar to (5%-phenyl)-methylpolysiloxane. High-purity helium (grade N6.0) was used as the carrier gas at a constant flow rate of 1.63 mL/min. The injector temperature was set at 280 °C, and the sample of the petroleum ether extract was injected in split mode at a volume of 1 μL. The GC oven temperature was first maintained at 60 °C for 5 min, then ramping linearly at 30 °C/min to 300 °C, and finally held constant at 300 °C for more 8 min. Mass spectrometry conditions were as follows: the ion source temperature was set at 200 °C and the interface at 300 °C. The solvent cut time was programmed for 2 min, with a scanning interval of 0.3 s. The instrument was set to scan from *m*/*z* 35 to 500, beginning after 3 min and ending at 19 min.

Using the NPClassifier, the identified chemical constituents were further classified according to their putative biosynthetic origins, major chemical superclasses, and specific compound classes.

### 3.6. Assessment of Total Phenolics, Flavonoid and Flavonol Compounds

#### 3.6.1. Total Phenolic Content

The phenolic content of the plant extracts was determined according to the polarity of each extract using Folin–Ciocalteu with slight modifications [45]. At first, 20 μL of plant extract (1 mg/mL) or different concentrations of gallic acid (standard) was added in each well of a 96-well microplate, followed by 100 μL of diluted Folin–Ciocalteu reagent (1:10 dilution). Subsequently, 75 μL of sodium 7.5% carbonate was added. The mixture was incubated at room temperature in the dark for 2 h. Then, the absorbance was recorded at 765 nm using a 96-well microplate reader (Perkin Elmer En Spire, Singapore). Total phenolic content was expressed as μg gallic acid equivalents (GAE) per mg of extract, Standard used is Gallic acid 0.5 mg/5 mL MeOH with a concentration range (25–200 µg/mL) number of replicates is *n* = 3. Calibration curve equation y = 0.0034x + 0.2205 and r^2^ = 0.962.

#### 3.6.2. Total Flavonoid Content

The total flavonoid content of the plant extracts was determined using a modified microplate assay originally described by Gnoyke et al. [45]. In brief, 50 µL of extract (1 mg/mL) was placed in a 96-well microplate along with 130 µL of methanol. Then, 10 µL of 1 M potassium acetate and 10 µL of 10% aluminum nitrate were added. The mixture was incubated at room temperature for 40 min to allow complex formation. The absorbance was then measured at 415 nm using a microplate spectrophotometer. Quercetin was used as a standard 0.5 mg/5 mL MeOH with a concentration range (25–200 µg/mL) number of replicates is *n* = 3, and results were expressed as μg quercetin equivalents (QE) per mg of extract. Calibration curve equation is y = 0.0048x and r^2^ = 0.999.

#### 3.6.3. Total Flavonol Content

Total flavonol content was measured according to the method described by Kumaran and Karunakaran [46], with slight modifications. In short, 50 µL of each extract (1 mg/mL) or standard solution was mixed with 50 µL of 2% aluminum chloride (prepared in distilled water) and 150 µL of 5% sodium acetate (*w*/*v*). The mixture was incubated at room temperature for 40 min. The absorbance was read at 400 nm using a microplate spectrophotometer. Quercetin was used as a reference standard 0.5 mg/5 mL MeOH with a concentration range (25–200 µg/mL) number of replicates is *n* = 3, and the flavonol content was expressed as µg QE/mg of extract. Calibration curve equation is y = 0.0071x + 0.0225 and r^2^ = 0.996.

### 3.7. Antioxidant Capacity

#### 3.7.1. DPPH Scavenging Capacity

The antioxidant potential of the extracts was determined based on their ability to scavenge the stable free radical (2,2-diphenyl-1-picrylhydrazyl) DPPH, following the method described by Blois [47]. A 0.1 mM solution of DPPH was prepared in methanol and adjusted to an absorbance of 0.5 nm at 517 nm. For the assay, 160 µL of the DPPH was mixed with 40 µL of each plant extract (4 mg/mL) at various concentrations. The reaction mixtures were incubated in the dark at room temperature for 30 min. Absorbance was then measured at 517 nm using a microplate reader (Perkin Elmer EnSpire, Singapore). Butylated hydroxyanisole (BHA) and butylated hydroxytoluene (BHT) were used as positive control. The percentage of DPPH radical scavenging activity was calculated using the following equation:

Scavenging activity (%) = [(A_control_ − A_sample_)/A_control_] × 100 (1)

where A_control_ and A_sample_ are the absorbance values of the control and the sample, respectively. A dose–response curve was plotted based on the percentage scavenging activity at various extract concentrations, allowing for the calculation of the concentration required to inhibit 50% of DPPH radical (i.e., IC_50_ value) and expressed in μg/mL.

#### 3.7.2. ABTS^•+^ Scavenging Capacity

The 2,2′-azinobis-(3-ethylbenzothiazoline)-6-sulfonic acid (ABTS^•+^) radical cation decolorization assay was conducted as described by Re et al. [48]. A 7 mM aqueous solution of ABTS^•+^ was mixed with 2.45 mM potassium persulfate and kept in the dark at room temperature for 16 h to generate the ABTS^•+^ radical cation. The ABTS^•+^ solution was then diluted with methanol to achieve an absorbance of 0.700 ± 0.020 at 734 nm. For the assay, 40 µL (4 mg/mL) of each extract (dissolved in methanol at various concentrations) was added to 160 µL of the ABTS^•+^ working solution in a 96-well microplate. The mixture was incubated in the dark for 10 min, and the absorbance was subsequently measured at 734 nm using a microplate reader. BHA and BHT were used as reference standards. The percentage of ABTS^•+^ radical scavenging activity was calculated using the following equation:

Scavenging activity (%) = [(A_control_ − A_sample_)/A_control_] × 100 (2)

where A_control_ and A_sample_ are the absorbance values of the control and the sample, respectively. The IC_50_ value of ABTS^•+^ assay was evaluated as described above for DPPH assay.

#### 3.7.3. Reducing Power Capacity

The reducing power (FRP) of the extracts (4 mg/mL) was determined following the method described by Oyaizu [49], based on their capacity to reduce ferric ions (Fe^3+^) to ferrous ions (Fe^2+^) in the potassium ferricyanide complex (K_3_Fe(CN)_6_) [34]. In each well of a 96-well plate, 10 μL of extract or standard (at different concentrations), 40 μL of phosphate buffer (0.2 M, pH 6.6), and 50 μL of 1% potassium ferricyanide solution were mixed. The mixtures were incubated at 50 °C for 20 min. After incubation, 50 μL of 10% trichloroacetic acid, 40 μL of distilled water, and 10 μL of 0.1% ferric chloride solution were added sequentially. The absorbance was measured at 700 nm using a microplate reader. BHA and ascorbic acid were used as positive controls. A dose–response curve was plotted based on the absorbance at various extract concentrations, that was used for the evaluation of the A_0.5_ value, indicating the extract concentration that caused an absorbance of 0.5 at 700 nm.

### 3.8. Anti-Inflammatory Activity

The in vitro anti-inflammatory activity was evaluated using a modified version of the Kandikattu method [35], which assesses the inhibition of protein denaturation. A lower percentage of protein denaturation indicates stronger anti-inflammatory activity because protein denaturation is considered a trigger for inflammation [50]. The modifications were made to optimize assay conditions for the tested extracts.

Briefly, 100 µL of each extract concentration (16 mg/mL) or standard was mixed with 100 µL of 0.4% bovine serum albumin (BSA) solution prepared in Tris-HCl buffer (pH 6.6). A white control (extract only) was prepared by mixing 100 µL of extract with 100 µL of Tris-HCl buffer. A negative control was prepared by mixing 100 µL of BSA solution with 100 µL of the solvent used to dissolve the extracts. The mixtures were incubated at 37 °C for 15 min, then heated in a water bath at 72 °C for 5 min. After cooling, turbidity was measured at 660 nm using a cuvette spectrophotometer. Diclofenac sodium was used as positive control.

The percentage inhibition of protein denaturation was calculated using the following equation:

Inhibition (%) = [(A_control_ − A_sample_)/A_control_] × 100 (3)

where A_control_ and A_sample_ are the absorbance values of the control (BSA + solvent, no inhibitor − complete denaturation) and the sample (BSA + extract), respectively.

### 3.9. Antibacterial Activity

Antibacterial activity was assessed using the standard agar disc diffusion method. All chemicals and products used in this study were purchased from Sigma-Aldrich (St. Louis, MO, USA). Four bacterial strains were used: one Gram-negative (*Escherichia coli* ATCC 25922) and three Gram-positive (*Staphylococcus aureus* ATCC 6538, *Staphylococcus epidermidis* ATCC 12228 and *Bacillus cereus* ATCC 14579). Bacterial inocula were prepared by transferring colonies from fresh cultures into 10 mL of sterile water, homogenizing with a vortex mixer, and adjusting turbidity to 0.5 McFarland standard.

The plant extract was dissolved in dimethyl sulfoxide (DMSO) (100 mg/mL) and diluted to concentrations of 12.5, 25 and 50 mg/mL. Wattman No. 01 filter paper discs (5 mm diameter) were sterilized at 120 °C for 20 min. Mueller-Hinton agar (MH) was poured into sterile Petri dishes to a depth of approximately 4 mm [51]. The bacterial suspension was evenly spread on the MH agar plates using a sterile swab. Sterile discs were soaked in the different concentrations of the plant extract and placed on the inoculated plates. Imipenem (100 μg/mL) was used as a positive control. Plates were pre-incubated at room temperature for 15 min to allow pre-diffusion, then incubated at 37 °C for 24 h. After incubation, the antibacterial activity was assessed by measuring the diameters of the inhibition zones surrounding the discs.

## 4. Conclusions

This study provides the first comprehensive phytochemical and biological investigation of *Convolvulus cantabrica* L., which was found to be rich in various potent bioactive compounds, such as flavonoids and phenolic acids, as demonstrated through both HPLC and GC-MS analyses.

GC–MS analysis of the petroleum ether fraction revealed a diverse profile, including fatty acids and esters (70.81%), sesquiterpenes and diterpenes (19.51%), fatty alcohols (6.02%), and other constituents. In contrast, the hydroalcoholic extract was richer in phenolic acids, as confirmed by HPLC. These compounds are known for their significant pharmacological potential, particularly their antioxidant and anti-inflammatory properties, which is consistent with the biological activities observed in the present study.

The plant extracts also exhibited antioxidant, anti-inflammatory, and antibacterial activities, which could be attributed to the presence of these bioactive compounds.

Antioxidant assays demonstrated that the ethyl acetate extract exhibited the strongest activity, while the anti-inflammatory capacity was also significant for the petroleum ether extract, suggesting that phenolic and flavonoid compounds are the main contributors to these effects.

Antibacterial tests further showed moderate inhibition against Gram-positive strains, depending on the solvent used, highlighting the selective sensitivity of the microorganisms and the influence of chemical composition on bioactivity.

These findings confirm the therapeutic potential of the genus *Convolvulus* and highlight its relevance as a source of natural bioactive compounds. The combined chemical and biological results reinforce the traditional use of this genus and support its potential applications in pharmacology, nutraceuticals, and natural product research.

However, further in-depth studies are necessary to isolate individual compounds, assess their toxicity profiles comprehensively, and explore a broader spectrum of biological activities. Future research should focus on detailed phytochemical investigations and pharmacological evaluations to fully realize the therapeutic potential of this species.

## Figures and Tables

**Table 1:** Weight and percentage yield of the extracts from *Convolvulus cantabrica*.

Extract	Weight (g)	Percentage Yield (%)
Hydroalcoholic extract	50 g	100%
Petroleum ether extract	2.43 g	4.86%
Ethyl acetate extract	11.20 g	22.40%
n-Butanol extract	30.00 g	60.00%

**Table 2:** Total phenolic, flavonoid and flavonol contents of the tested extracts.

Extract	Total Phenolic Content (µg GAE/mg)	Total Flavonoid Content (µg QE/mg)	Total Flavonol Content (µg QE/mg)
Hydro-alcoholic	193.872 ± 4.898 ^c^	65.277 ± 1.915 ^c^	32.253 ± 0.365 ^c^
Petroleum ether	67.009 ± 4.727 ^d^	71.527 ± 0.294 ^c^	95.328 ± 1.129 ^b^
Ethyl acetate	606.421 ± 4.014 ^a^	363.75 ± 2.504 ^a^	127.441 ± 1.438 ^a^
n-Butanol	414.460 ± 2.795 ^b^	221.527 ± 7.807 ^b^	93.075 ± 1.424 ^b^

All values are expressed as mean ± SD (*n* = 3). Statistical differences between groups were examined with Tukey’s test. Means sharing the same letter are not significantly different at *p* > 0.05.

**Table 3:** Phenolic compounds identified in Hydroalcoholic extract by HPLC-DAD. Calibration curve was obtained with 1, 5, 10, 20, 50, 70, 100 ppm concentration.

No	Phenolic Compound	r^2^	Wavelength (nm)	Retention Time (min)	Concentration (ppm)
1	3-Hydroxy Benzoic Acid	0.99928	287	22.545	N.D.
2	4-Hydroxy Benzoic Acid	0.99994	278	17.647	N.D.
3	Benzoic Acid	0.99986	278	47.629	N.D.
4	Catechin Hydrate	0.99906	278	11.499	N.D.
5	Chlorogenic Acid	0.9997	330	16.239	153.765
6	Caffeic Acid	0.99892	330	21.476	33.047
7	Epicatechin	0.99879	278	20.169	N.D.
8	Gallic Acid	0.99966	278	5.912	1.099
9	Hesperidin	0.99705	287	65.989	7.459
10	p-Coumaric Acid	0.99982	330	33.597	N.D.
11	Quercetin	0.99962	254	76.313	3.047
12	Rosmarinic Acid	0.99907	330	70.655	161.897
13	Sinnapic Acid	0.99925	330	37.264	N.D.
14	Syringic Acid	0.99839	278	22.628	N.D.
15	trans-Cinnamic Acid	0.99998	278	75.207	3.165
16	trans-Ferulic Acid	0.99993	330	37.202	N.D.

r^2^ correlation coefficient. N.D. Not Detected. ppm mg/kg—mg/L.

**Table 4:** Compounds identified by GC-MS analysis in petroleum ether extract.

Family Concentration	Peak	Compound Name	Chemical Formula	Retention TIME (min)	Area	Area%
**Fatty Acids and Esters** **Total: 70.81%**	32	n-Hexadecanoic acid	C_16_H_32_O_2_	40.048	12,820,761	17.10
	43	10(E),12(Z)-Conjugated linoleic acid	C_18_H_32_O_2_	45.622	2,495,049	3.33
	18	Tetradecanoic acid	C_14_H_28_O_2_	34.288	183,737	0.25
	23	Pentadecanoic acid	C_15_H_30_O_2_	36.898	115,774	0.15
	36	Heptadecanoic acid	C_17_H_34_O_2_	43.170	110,629	0.15
	5	Nonanoic acid	C_9_H_18_O_2_	19.610	88,090	0.12
	8	Dodecanoic acid	C_12_H_24_O_2_	28.801	79,556	0.11
	4	Hexanoic acid	C_6_H_12_O_2_	9.423	48,663	0.06
	34	Hexadecanoic acid, ethyl ester	C_18_H_36_O_2_	40.962	14,519,626	19.36
	46	9,12,15-Octadecatrienoic acid, ethyl ester, (Z,Z,Z)-	C_20_H_34_O_2_	46.822	7,753,648	10.34
	45	Linoleic acid ethyl ester	C_20_H_36_O_2_	46.591	6,961,703	9.28
	39	9,12-Octadecadienoic acid (Z,Z)-, methyl ester	C_19_H_34_O_2_	44.233	2,619,459	3.49
	48	Octadecanoic acid, ethyl ester	C_20_H_40_O_2_	47.848	1,812,528	2.42
	40	6-Octadecenoic acid, methyl ester, (Z)-	C_19_H_36_O_2_	44.457	1,802,698	2.40
	33	Ethyl 9-hexadecenoate	C_18_H_34_O_2_	40.749	568,798	0.76
	29	Palmitate <methyl->	C_17_H_34_O_2_	38.797	502,335	0.67
	47	(E)---9-Octadecenoic acid ethyl ester	C_20_H_38_O_2_	47.038	290,846	0.39
	42	Methyl stearate	C_19_H_38_O_2_	45.421	211,456	0.28
	27	Pentadecanoic acid, ethyl ester	C_17_H_34_O_2_	37.856	115,221	0.15
**Alkanes and Alkenes** **0.69%**	31	1-(1,2-dimethylpropyl)-1-methyl-2-nonyl-Cyclopropane	C_18_H_36_	39.607	77,726	0.10
	20	1-Nonadecene	C_19_H_38_	35.096	243,353	0.32
	11	1-Heptadecene	C_17_H_34_	29.622	109,282	0.15
	37	Henicos-1-ene	C_21_H_42_	43.496	90,287	0.12
**Sesquiterpene and Diterpenes 19.51%**	9	(−)-Spathulenol	C_15_H_24_O	29.207	221,858	0.30
	17	(1R,7S,E)-7-Isopropyl-4,10-dimethylenecyclodec-5-enol	C_15_H_24_O	32.269	137,863	0.18
	10	Caryophyllene oxide (Isomer 1)	C_15_H_24_O	29.345	245,624	0.33
	12	Muurola-4,10(14)-dien-1.beta.-ol	C_15_H_24_O	30.656	69,215	0.09
	13	Caryophylla-4(12),8(13)-dien-5.alpha.-ol	C_15_H_24_O	30.880	86,515	0.12
	16	Caryophyllene oxide (Isomer 2)	C_15_H_24_O	31.865	136,360	0.18
	19	(1R,2R,4S,6S,7S,8S)-8-Isopropyl-1-methyl-3-methylenetricyclo[4.4.0.02,7]decan-4-ol	C_15_H_24_O	34.820	375,523	0.50
	30	1,3,6,10-Cyclotetradecatetraene, 3,7,11-trimethyl-14-(1-methylethyl)-,[S-(E,Z,E,E)]	C_20_H_32_	38.925	1,735,542	2.31
	35	4-Isopropyl-1,7,11-trimethyl-2,7,11-cyclotetradecatrien-1-ol	C_20_H_34_O	42.694	6,786,538	9.05
	41	Phytol	C_20_H_40_O	44.909	4,705,883	6.28
	21	Neophytadiene	C_20_H_38_	36.281	130,630	0.17
**Phenolic and** **Aromatics 1.64%**	6	Carvacrol	C_10_H_14_O	20.680	60,148	0.08
	28	Ethyl (E)-ferulate	C_12_H_14_O_4_	38.525	104,414	0.14
	24	1,2-Benzenedicarboxylic acid, bis(2-methylpropyl) ester	C_16_H_22_O_4_	37.049	188,845	0.25
	3	methyl Benzene	C_7_H_8_	3.456	698,881	0.93
	15	1-(1,5-dimethyl-4-hexenyl)-4-methyl-Benzene	C_15_H_22_	31.478	179,012	0.24
**Fatty Alcohols** **6.02%**	44	9,12,15-Octadecatrien-1-ol	C_18_H_32_O	45.852	3,975,903	5.30
	26	1-Hexadecanol	C_16_H_34_O	37.435	277,244	0.37
	38	n-Nonadecanol-1	C_19_H_40_O	43.895	266,183	0.35
**Ketones** **0.77%**	22	6,10,14-trimethyl-2-Pentadecanone,	C_18_H_36_O	36.434	489,163	0.65
	25	10-Nonadecanone	C_19_H_38_O	37.241	89,959	0.12
**Others** **0.55%**	14	Jasmonate <methyl-, epi->	C_13_H_20_O_3_	31.369	100,215	0.13
	1	Hydroxyacetic acid, hydrazide	C_2_H_6_N_2_O_2_	2.047	101,921	0.14
	2	2,2-Dimethoxybutane	C_6_H_14_O_2_	3.343	32,451	0.04
	7	5,6,7,7a-tetrahydro-4,4,7a-trimethyl-,(R)-2(4H)-Benzofuranone	C_11_H_16_O_2_	27.698	176,428	0.24
**Total**					**74,993,543**	**99.99**

**Table 5:** Antioxidant capacity as assessed by DPPH, ABTS^•+^ and reducing power assays.

Extracts	DPPH	ABTS^•+^	Reducing Power
	IC_50_ (µg/mL)		A_0.5_ (µg/mL)
Hydroalcoholic	48.73 ± 1.31 ^b^	28.74 ± 4.99 ^b^	40.68 ± 1.41 ^b^
Petroleum ether	>800 ^a^	548.43 ± 65.51 ^a^	>200 ^a^
Ethyl acetate	13.60 ± 1.30 ^d^	7.26 ± 0.01 ^c^	14.89 ± 0.90 ^d^
n-Butanol	17.69 ± 1.17 ^c^	6.90 ± 0.18 ^c^	23.14 ± 0.60 ^c^
**Positive controls**			
BHT	22.32 ± 1.19 ^c^	1.29 ± 0.30 ^d^	-
BHA	5.73 ± 0.41 ^e^	1.81 ± 0.10 ^d^	-
Ascorbic acid	-	-	6.77 ± 1.15 ^e^
α-Tocopherol	-	-	34.93 ± 2.38 ^b^

All values are expressed as mean ± SD (*n* = 3). Statistical differences between groups were examined with Tukey’s test. Means sharing the same letter are not significantly different at *p* > 0.05.

**Table 6:** Anti-inflammatory activity.

Extracts	IC_50_ (µg/mL)
Petroleum ether	419.30 ± 4.48 ^c^
Ethyl acetate	3172.65 ± 35.05 ^c^
**Positive control**	
Diclofenac sodium	87.25 ± 0.19

All values are expressed as mean ± SD (*n* = 3). ^c^ *p* < 0.001 statistically significant different from diclofenac sodium.

**Table 7:** Zones of inhibition of *Convolvulus cantabrica* L. extracts.

Bacteria	Conc. of Extract (mg/mL)	Mean Zone of Inhibition (mm)					
		Hydroalcoholic	Petroleum Ether	Ethyl Acetate	n-Butanol	Imipenem (100 μg/mL)	Negative Control (DMSO)
*Staphylococcus* *aureus*	100	0	0	0	0	50	0
	50	0	0	0	0		
	25	0	0	0	0		
	12.5	0	0	0	0		
	6.25	0	0	0	-		
*Bacillus cereus*	100	10.5 ± 0.7	9 ± 1.41	10.5 ± 0.7	0	40.1	0
	50	07.5 ± 0.7	7.5 ± 0.7	9.5 ± 0.7	0		
	25	7.5 ± 0.7	6.5 ± 0.7	8.5 ± 0.7	0		
	12.5	6.5 ± 0.7	6.5 ± 0.7	7 ± 1.41	0		
	6.25	0	0	0	-		
*Escherichia coli*	100	0	0	0	0	20.7	0
	50	0	0	0	0		
	25	0	0	0	0		
	12.5	0	0	0	0		
	6.25	0	0	0	-		
*Staphylococcus* *epidermidis*	100	0	0	0	0	30.3	0
	50	0	0	0	0		
	25	0	0	0	0		
	12.5	0	0	0	0		
	6.25	0	0	0	-		

‘0’ indicates absence of an inhibition zone.

## Data Availability

Data is contained within the article.

## Supplementary Materials

The following supporting information can be downloaded at: https://www.mdpi.com/article/10.3390/molecules31010058/s1, Figure S1. HPLC–DAD chromatogram of the hydroalcoholic extract. The calibration curve was established using standard concentrations of 1, 5, 10, 20, 50, 70, and 100 ppm. Figure S2. Percentage distribution of chemical families identified by GC–MS analysis.

## Abbreviations

ABTS	2,2′-azino-bis(3-ethyl- benzothiazoline-6-sulfonic acid
BHA	butylated hydroxyanisole
BHT	butylated hydroxytoluene
DMSO	dimethyl sulfoxide
DPPH	2,2-diphenyl-1-picrylhydrazyl
EA	ethyl acetate
FRAP	ferric reducing antioxidant power assay
MH	Mueller-Hinton Agar
n-Bu	n-butanol
PE	petroleum ether

## References

[1] Kurhekar J.V., Egbuna C., Mishra A.P., Goyal M.R. (2021). Chapter 4—Ancient and Modern Practices in Phytomedicine. Preparation of Phytopharmaceuticals for the Management of Disorders.

[2] Lin Y.-W., Yang F.-J., Chen C.-L., Lee W.-T., Chen R.-S. (2010). Free Radical Scavenging Activity and Antiproliferative Potential of *Polygonum cuspidatum* Root Extracts. J. Nat. Med..

[3] Alvarez-leite J.I. (2025). The Role of Bioactive Compounds in Human Health and Disease. Nutrients.

[4] Peng Y., Li Y., Yang Y., Gao Y., Ren H., Hu J., Cui X., Lu W., Tao H., Chen Z. (2022). The Genus *Porana* (Convolvulaceae)—A Phytochemical and Pharmacological Review. Front. Pharmacol..

[5] Umekar M.J. (2021). Convolvulaceae: A Morning Glory Plant. Int. J. Pharm. Sci. Rev. Res..

[6] da Mata R.R., Oliveira R.R., Pereira S.G., Rocha T.L., Castro Aguiar J.V., da Silva-Matos R.R.S. (2025). Phytochemical Analysis of Secondary Metabolites from the Flowers and Roots of the Plant *Ipomoea asarifolia*. Nat. Prod. Res..

[7] Furtado A.A., Torres-Rêgo M., Lima M.C.J.S., Bitencourt M.A.O., Estrela A.B., Souza da Silva N., da Silva Siqueira E.M., Tomaz J.C., Lopes N.P., Silva-Júnior A.A. (2016). Aqueous Extract from *Ipomoea asarifolia* (Convolvulaceae) Leaves and Its Phenolic Compounds Have Anti-Inflammatory Activity in Murine Models of Edema, Peritonitis and Air-Pouch Inflammation. J. Ethnopharmacol..

[8] Tani C., Bourgeois T., Munoz F. (2010). Floristic Aspects of Weed Flora of the Oranian Phytogeographic Territory (North-West Algeria) and Persistency of Rare and/or Endemic Species. Flora Mediterr..

[9] Chen G.-T., Lu Y., Yang M., Li J.-L., Fan B.-Y. (2018). Medicinal Uses, Pharmacology, and Phytochemistry of Convolvulaceae Plants with Central Nervous System Efficacies: A Systematic Review. Phytother. Res..

[10] Simões A.R.G., Eserman L.A., Zuntini A.R., Chatrou L.W., Utteridge T.M.A., Maurin O., Rokni S., Roy S., Forest F., Schneeweiss G.M. (2022). A Bird’s Eye View of the Systematics of Convolvulaceae: Novel Insights From Nuclear Genomic Data. Front. Plant Sci..

[11] Romeiro L.d.A., da Silva E.F., Vasconcelos L.V., Lopes K.d.S., Carreira L.M.M., Guimarães J.T.F. (2023). Pollen Morphology of Convolvulaceae from Southeastern Amazonian Cangas and Its Relevance for Interaction Networks and Paleoenvironmental Studies. Plants.

[12] Evert R.F., Eichhorn S.E. (2013). Raven Biology of Plants.

[13] Eich E. (2008). Solanaceae and Convolvulaceae: Secondary Metabolites.

[14] Simões A.R.G., Huerta-Ramos G., Moreira A.L.C., Paz J.R.L., Grande Allende J., Pisuttimarn P., Rattanakrajang P., Barbosa J.C.J., Simão-Bianchini R., Kojima R.K. (2024). Sweet Potato, Morning Glories, Bindweeds: An Overview of Convolvulaceae. Rheedea.

[15] Aykurt C. (2018). Taxonomic Revision of the Genus *Convolvulus* L. (Convolvulaceae) in Turkey. Biol. Divers. Conserv..

[16] Osman E.E.A., Shemis M.A., Abdel-Hameed E.S.S., Gouda A.E., Hassan H., Atef N., Mamdouh S. (2024). Phytoconstituent Analysis, Anti-Inflammatory, Antimicrobial and Anticancer Effects of Nano Encapsulated *Convolvulus arvensis* L. Extracts. BMC Complement. Med. Ther..

[17] Al-snafi P.A.E., Medicine C. (2016). The Chemical Constituents and Pharmacological Effects of *Convolvulus arvensis* and *Convolvulus scammonia*—A Review II. Plants Profile.

[18] Balkrishna A., Thakur P., Varshney A. (2020). Phytochemical Pro Fi Le, Pharmacological Attributes and Medicinal Properties of Convolvulus Prostratus—A Cognitive Enhancer Herb for the Management of Neurodegenerative Etiologies. Front. Pharmacol..

[19] Salehi B., Krochmal-Marczak B., Skiba D., Patra J.K., Das S.K., Das G., Popović-Djordjević J.B., Kostić A.Ž., Anil Kumar N.V., Tripathi A. (2020). *Convolvulus* Plant—A Comprehensive Review from Phytochemical Composition to Pharmacy. Phytother. Res..

[20] Salamatullah A.M. (2022). *Convolvulus Arvensis*: Antioxidant, Antibacterial, and Antifungal Properties of Chemically Profiled Essential Oils: An Approach Against Nosocomial Infections. Life.

[21] EsmaHassler M., Muer T. (2022). Flora Germanica: Alle Farn- und Blütenpflanzen Deutschlands in Text und Bild.

[22] Del Rio D., Rodriguez-Mateos A., Spencer J.P.E., Tognolini M., Borges G., Crozier A. (2013). Dietary (Poly)phenolics in Human Health: Structures, Bioavailability, and Evidence of Protective Effects. Antioxid. Redox Signal..

[23] Al-Rifai A., Aqel A., Al-Warhi T., Wabaidur S.M., Al-Othman Z.A., Badjah-Hadj-Ahmed A.Y. (2017). Antibacterial, Antioxidant Activity of Ethanolic Plant Extracts of Some *Convolvulus* Species and Their DART-ToF-MS Profiling. Evid. Based. Complement. Alternat. Med..

[24] Özay C., Mammadov R. (2019). Antioxidant Activity, Total Phenolic, Flavonoid and Saponin Contents of Different Solvent Extracts of *C. Phrygius* Bornm. Curr. Perspect. Med. Aromat. Plants.

[25] Guan H., Luo W., Bao B., Cao Y., Cheng F., Yu S., Fan Q., Zhang L., Wu Q., Shan M. (2022). A Comprehensive Review of Rosmarinic Acid: From Phytochemistry to Pharmacology and Its New Insight. Molecules.

[26] Actions M., Potentials T. (2024). Chlorogenic Acid: A Systematic Review on the Biological Functions, Mechanistic Actions, and Therapeutic Potentials. Nutrients.

[27] Sciences M. (2023). Caffeic Acid and Diseases—Mechanisms of Action. Int. J. Mol. Sci..

[28] Bingol Z., Kızıltaş H., Gören A.C., Kose L.P., Topal M., Durmaz L., Alwasel S.H., Gulcin İ. (2021). Antidiabetic, Anticholinergic and Antioxidant Activities of Aerial Parts of Shaggy Bindweed (*Convulvulus betonicifolia* Miller Subsp.)—Profiling of Phenolic Compounds by LC-HRMS. Heliyon.

[29] Cengiz S., Mammadov R., Aykurt C., Taşdelen G. (2015). Variations in Antioxidant Enzyme Levels of Rats Exposed to Etha-nol Extracts of *Convolvulus* Species. Ind. Crops Prod..

[30] Noori M., Bahrami B., Mousavi A., Khalighi A., Jafari A. (2016). Flower Flavonoids of *Convolvulus* L. species in Markazi Province, Iran. Asian J. Plant Sci..

[31] Shoker R.M.H., Jawad A.M. (2013). Evaluation of Isolated Compounds Activity from *Convolvulus arvensis* Against Algae. Iraqi J. Sci..

[32] Miri Y. (2025). Ben Essential Oils: Chemical Composition and Diverse Biological Activities: A Comprehensive Review. Nat. Prod. Commun..

[33] Osorio S., Vallarino J.G. (2019). From Central to Specialized Metabolism: An Overview of Some Secondary Compounds Derived from the Primary Metabolism for Their Role in Conferring Nutritional and Organoleptic Characteristics to Fruit. Front. Plant Sci..

[34] Hrichi S., Chaâbane-Banaoues R., Alibrando F., Altemimi A.B., Babba O., Majdoub Y.O.E., Nasri H., Mondello L., Babba H., Mighri Z. (2022). Chemical Composition, Antifungal and Anti-Biofilm Activities of Volatile Fractions of *Convolvulus althaeoides* L. Roots from Tunisia. Molecules.

[35] Hassine M., Znati M., Flamini G., Ben H. (2014). Chemical Composition, Antibacterial and Cytotoxic Activities of the Essential Oil from the Flowers of Tunisian *Convolvulus althaeoides* L. Nat. Prod. Res. Former. Nat. Prod. Lett..

[36] Sanchez-Moreno C. (2002). Review: Methods Used to Evaluate the Free Radical Scavenging Activity in Foods and Biological Systems. Food Sci. Technol. Int.—Food Sci Technol. Int..

[37] Menezes A., Filho D.A., Gil E.D.S., Oliveira R. (2020). Correlation of Polyphenol Content and Antioxidant Capacity of Selected Teas and Tisanes from Brazilian Market. Braz. J. Food Technol..

[38] Han R., Zhang J., Skibsted L.H. (2012). Reaction Dynamics of Flavonoids and Carotenoids. Molecules.

[39] Thakral J., Borar S., Roopa, Kalia A.N. (2010). Antioxidant Potential Fractionation from Methanol Extract of Aerial Parts of *Convolvulus arvensis* Linn. (Convolvulaceae). Int. J. Pharm. Sci. Drug Res..

[40] Lü J.-M., Lin P.H., Yao Q., Chen C. (2010). Chemical and Molecular Mechanisms of Antioxidants: Experimental Approaches and Model Systems. J. Cell. Mol. Med..

[41] Awaad A., Al-Rifai A., El-Meligy R., Alafeefy A., Alqasoumi S. (2014). Antiulcerogenic Activity of Convolvulus Species. Austin Chromatogr..

[42] Gupta G.L., Fernandes J. (2019). Protective Effect of *Convolvulus pluricaulis* Against Neuroinflammation Associated Depressive Behavior Induced by Chronic Unpredictable Mild Stress in Rat. Biomed. Pharmacother..

[43] Lu Y.-H., Hong Y., Zhang T.-Y., Chen Y.-X., Wei Z.-J., Gao C.-Y. (2022). Rosmarinic Acid Exerts Anti-Inflammatory Effect and Relieves Oxidative Stress via Nrf2 Activation in Carbon Tetrachloride-Induced Liver Damage. Food Nutr. Res..

[44] Ayad R., Keskinkaya H.B., Atalar M.N., Lefahal M., Zaabat N., Makhloufi E.H., Demirtas I., Trifa W., Akkal S., Medjroubi K. (2023). *Jurinea humilis* DC. Polar Extract: HPLC Analysis, Photoprotective, Antioxidant Activities and Bioactive Content. Chem. Afr..

[45] Gnoyke S., Popken A., Böhm V. (2010). Antioxidant Capacity and Related Parameters of Different Fruit Formulations. Lwt—Food Sci. Technol..

[46] Kumaran A., Karunakaran R.J. (2007). In Vitro Antioxidant Activities of Methanol Extracts of Five *Phyllanthus* Species from India. LWT—Food Sci. Technol..

[47] Baliyan S., Mukherjee R., Priyadarshini A., Vibhuti A., Gupta A., Pandey R.P., Chang C. (2022). Determination of Antioxidants by DPPH Radical Scavenging Activity and Quantitative Phytochemical Analysis of *Ficus religiosa*. Molecules.

[48] Re R., Pellegrini N., Proteggente A., Pannala A., Yang M., Rice-Evans C. (1999). Antioxidant Activity Applying an Improved ABTS Radical Cation Decolorization Assay. Free Radic. Biol. Med..

[49] Oyaizu M. (1986). Studies on Products of Browning Reaction. Jpn. J. Nutr. Diet..

[50] Karthik K.K.K., Kumar P.B.R., Priya R.V., Kumar K.S., Rathore R.S.B. (2013). Evaluation of Anti-Inflammatory Activity of *Canthium parviflorum* by In-Vitro Method. Indian J. Res. Pharm. Biotechnol..

[51] Alnamer R., Alnamer R., Alaoui K., Doudach L., Bouidida E.H., Al-sobarry M., Benjouad A., Cherrah Y. (2012). Investigation of Methanolic and Aqueous Extract of *Lavandula officinalis* for Toxicity and Antibacterial Activity. World J. Pharm. Res..

