# Supplementary material for: Phytochemical Profile of Convolvulus cantabrica Extracts and Their Biological Activity
Source: Molecules. 2025 Dec 23;31(1):58. doi: 10.3390/molecules31010058 (PMC12787251; doi:10.3390/molecules31010058)
Supplement: Supplementary file 1 [file molecules-31-00058-s001.zip › molecules-3990679-supplementary.pdf]

## Supplementary Materials

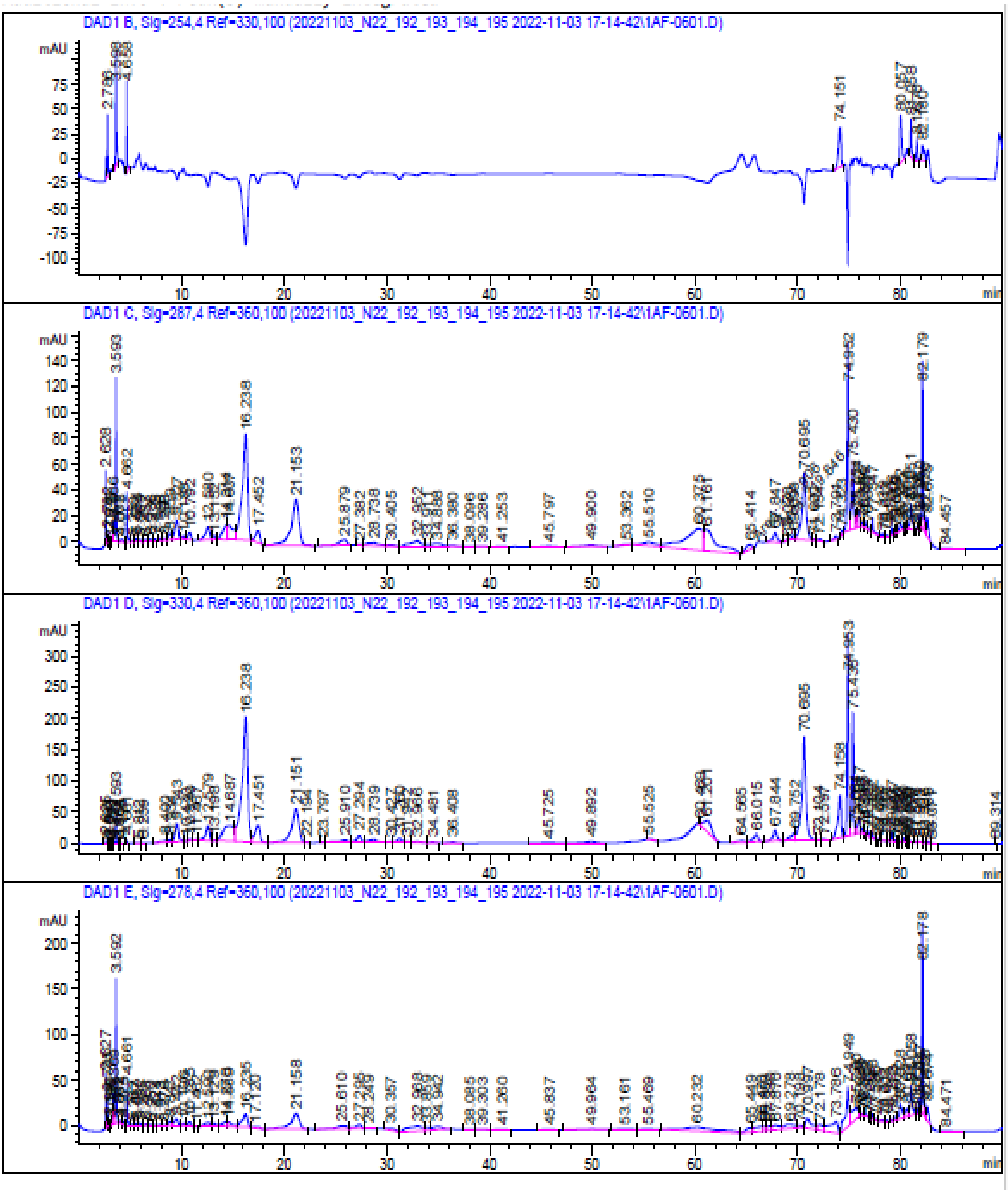


**Figure S1.** Chromatogram of Hydroalcoholic extract by HPLC-DAD. Calibration curve was obtained with 1, 5, 10, 20, 50, 70, 100 ppm concentration

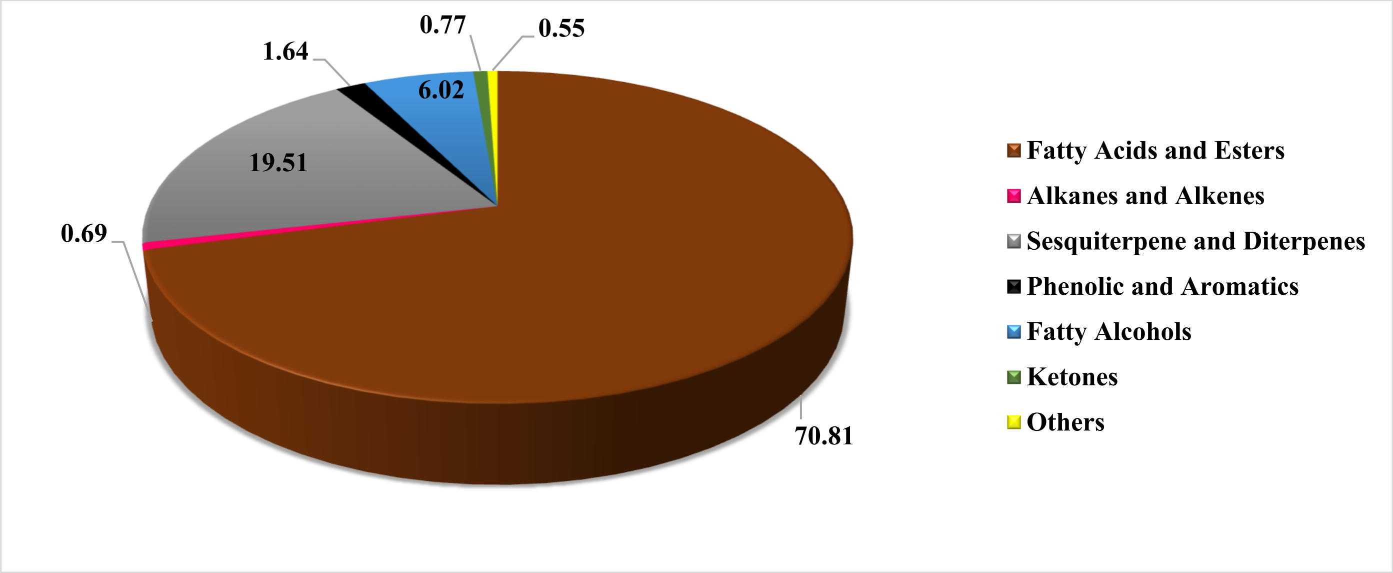


**Figure S2.** Percentage of the chemical families identified by GC-MS analysis.
